# Follow-Up Findings in Multiple System Atrophy from [^123^I]Ioflupane Single-Photon Emission Computed Tomography (SPECT): A Prospective Study

**DOI:** 10.3390/biomedicines11112893

**Published:** 2023-10-26

**Authors:** Javier Villena-Salinas, Simeón José Ortega-Lozano, Tomader Amrani-Raissouni, Eduardo Agüera, Javier Caballero-Villarraso

**Affiliations:** 1 Nuclear Medicine Service, Virgen de la Victoria University Hospital, 29010 Málaga, Spain; villenajavier@outlook.es (J.V.-S.); simeon.ortega.sspa@juntadeandalucia.es (S.J.O.-L.); tomader.amrani.sspa@juntadeandalucia.es (T.A.-R.); 2 Neurology Service, Reina Sofia University Hospital, 14004 Córdoba, Spain; doctoredu@gmail.com; 3 Maimónides Biomedical Research Institute of Córdoba (IMIBIC), 14004 Córdoba, Spain; 4 Clinical Analyses Service, Reina Sofía University Hospital, 14004 Córdoba, Spain; 5 Department of Biochemistry and Molecular Biology, Universidad of Córdoba, 14071 Córdoba, Spain

**Keywords:** multiple system atrophy, dysautonomia, follow-up study, diagnostic accuracy, functional neuroimaging testing, Ioflupane-123

## Abstract

Background: Multiple system atrophy (MSA) is subdivided into two types: MSA-P (parkinsonian) and MSA-C (cerebellar). Brain SPECT allows for the detection of nigrostriatal involvement, even in the early stages. To date, the scientific literature does not show a consensus on how to follow-up MSA, especially MSA-C. Our aim was to analyze the diagnostic effectiveness of repeat [^123^I]Ioflupane SPECT for the follow-up of MSA. Methods: A longitudinal observational study on 22 MSA patients (11 males and 11 females). Results: Significant changes were obtained in the quantitative SPECT assessments in the three Striatum/Occipital indices. The qualitative SPECT diagnosis did not show differences between the initial and evolving SPECT, but the neurologist’s clinical suspicion did. Our results showed a brain deterioration of around 31% at 12 months, this being the optimal cut-off for differentiating a diseased subject (capable of solving diagnostic error rate). Previous imaging tests were inconclusive, as they showed less deterioration in the SPECT and quantitative assessments with respect to the group of confirmed patients. Repeated SPECT increased the diagnostic sensitivity (50% vs. 75%) and positive predictive value (72.73% vs. 77%). In addition, repeated SPECT proved decisive in the diagnosis of initial inconclusive cases. Conclusion: Repeat SPECT at 12 months proves useful in the diagnosis and follow-up of MSA.

## 1. Introduction

Multiple system atrophy (MSA) is a neurodegenerative disease with a fatal course that occurs sporadically in adults older than 30 years [1,2]. This entity is subdivided into MSA-P if parkinsonian symptoms predominate or MSA-C when cerebellar involvement predominates. This categorization varies depending on the clinical course of the patient [3]. There is a predominance of MSA-P in Europe and the USA and MSA-C in Asia. However, in Spain, there is a majority of MSA-C [4,5,6,7].

Following the revision of the diagnostic criteria for MSA in 2008, a positive SPECT result with [^123^I]Ioflupane [3] was included as possible support for MSA-C. This test allows for the detection of nigrostriatal pathway involvement in vivo and noninvasively. However, it seems that it was not taken into account in the recent revision of its diagnostic criteria [8]. 

MSA cases show an asymmetric loss of putamen nucleus function, similar to idiopathic Parkinson’s disease (PD) but with a greater involvement of the head of the caudate nucleus [9]. Other studies have shown a greater decrease in presynaptic dopaminergic function in both striatal nuclei, rapidly and symmetrically in MSA compared to PD [10]. In studies performed using PET (Positron Emission Tomography) with another similar radiotracer, it was observed that MSA-P patients showed a greater involvement of the nigrostriatal pathway compared to MSA-C patients (who had a more uniform pattern). In addition, in some cases of MSA-C, the test was normal [11]. This coincides with early survival studies, which have postulated a more rapid functional deterioration in MSA-P patients compared to MSA-C patients [4].

Recently, a meta-analysis was performed that included 35 studies with a total of 701 patients: 356 with MSA-P, 62 with MSA-C, 204 with PSP (progressive supranuclear palsy), and 79 with DCB (corticobasal degeneration). A greater striatal involvement was observed in the PSP cases than in the PD and MSA-P cases, as well as in MSA-P patients with respect to MSA-C patients [12]. In an evaluation of 30 patients with MSA-C (possible or probable) without parkinsonism, signs of subclinical nigrostriatal dopaminergic deficit were found in [^123^I]Ioflupane SPECT. No relationship was established between such involvement and the duration of the disease, either with cerebellar dysfunction or pontine atrophy on MRI [13].

Given that a normal study cannot exclude a diagnosis of MSA and that, in addition, MSA-C can show an apparent normality in the density of presynaptic dopamine transporters in a visual analysis and/or borderline values in a semiquantitative analysis, it is particularly relevant to perform follow-up studies in both cases. All this, added to the rapid progression of MSA, would allow for its evolution to be monitored using [^123^I]Ioflupane SPECT [14]. In this regard, while serial studies on patients with PD do not seem to be justified, they would be on patients with suspected MSA, and especially those with MSA-C in its early stages [13,15], although these proposals have not been sufficiently contrasted. In fact, a recent article studied 7 patients with MSA-C, 5 with MSA-P, and 18 with PD, concluding that [^123^I]Ioflupane SPECT and a semiquantitative analysis with SBR (Specific Binding Ratio) constitutes a specific biomarker of the progression of this disease, as well as of the subtype of dopaminergic degeneration in both MSA and PD [16].

To date, the scientific literature has not shown a consensus on how to follow-up MSA, especially MSA-C. Our aim is to understand the variations shown by these neuroimaging studies on MSA patients as time progresses. This way, the identification of possible changes typical of the progression of this disease could reveal the diagnostic effectiveness of [^123^I]Ioflupane SPECT for the follow-up of MSA, and therefore the appropriateness of performing an evolutionary neuroimaging study on these patients.

## 2. Materials and Methods

This was a single-center longitudinal prospective observational study. Data were collected from 22 patients with MSA (11 males and 11 females) who underwent a second [^123^I]Ioflupane SPECT during the follow-up period. 

The SPECT study was performed with a Siemens^®^ gamma camera, (Erlangen, Germany) model SymbiaTM, Symbia^®^ model, equipped with a dual head. A low-energy, high-resolution collimator was used. The results were evaluated qualitatively with a visual analysis and quantitatively by using predefined Regions of Interest (ROIs) to obtain Striatum/Occipital indices for each striatal nucleus (right S/O and left S/O) and globally (global S/O), the latter being the arithmetic mean of both [17]. The reference area of non-specific uptake was the occipital lobe [18].

### 2.1. Ethical Considerations

The authorization for this study was obtained from our reference Biomedical Research Ethics Committee. For inclusion in the study, informed consent was obtained from each patient. 

### 2.2. Statistical Study

In the descriptive study, qualitative variables were collected as absolute and percentage frequencies, as well as the mean and standard deviation in the quantitative variables, since they presented a normal distribution after applying the Shapiro–Wilks test. To calculate the association between the qualitative variables, the Chi-square test with Fisher’s correction was applied. To analyze the baseline and final differences in the quantitative variables, the paired Student’s *t*-test (parametric) or Wilcoxon’s *t*-test (nonparametric) were used. Spearman’s correlation coefficient was applied between the continuous quantitative variables. A value of *p* < 0.05 was considered to be significant.

## 3. Results

The demographic, clinical, and diagnostic variables are shown in Table 1. The mean age was 65 ± 9 years (ranging from 47 to 77 years). The mean time under study was 26 months.

The quantitative results of the baseline SPECT and repeated (or evolving) SPECT are shown in Table 2. While in the baseline SPECT there were 18.2% inconclusive results, in the evolving SPECT, there were no inconclusive cases.

Diagnostic accuracy values were calculated for both scans (baseline and evolving), taking as a reference the diagnosis at the end of the study period, since no postmortem confirmatory study was available (Table 3). When performing the second SPECT during the patients’ follow-ups, it could be observed how the diagnostic performance increased, especially in terms of sensitivity and predictive values.

When looking for quantitative changes in [^123^I]Ioflupane uptake over time (baseline and final), significant differences were observed in the three S/O indices. Global S/O (Striatum/Occipital): $\bar{x}$ = 1.4 (SD = 0.3) vs. $\bar{x}$ = 1.3 (SD = 0.2), *p* = 0.025); right S/O: $\bar{x}$ = 1.4 (SD = 0.3) vs. $\bar{x}$ = 1.3 (SD = 0.2), *p* = 0.031); and left S/O: $\bar{x}$ = 1.4 (SD = 0.2) vs. $\bar{x}$ = 1.3 (SD = 0.2), *p* = 0.021.

To determine the possible differences between cerebral hemispheres (possible changes in dominance according to evolution), the degrees of deterioration of both the right and left striatal nuclei were calculated. The basal versus final differences in each of the striatal nuclei were compared. The results showed that both striate nuclei deteriorated statistically significantly over time (*p* < 0.05). When comparing the degrees of deterioration of both hemispheres to find out if one of them deteriorated faster than the other, no significant differences were found.

Similarly, in order to detect the possible changes in the qualitative (visual) interpretations of the [^123^I]Ioflupane SPECT results over time, we analyzed the possible changes in the qualitative diagnoses established by the nuclear medicine physicians according to the baseline study and evolutionary study, and found no significant differences (*p* = 0.188). Likewise, there was a significant association between the initial and final diagnostic suspicion (*p* < 0.001).

In 13 patients (59.09%), the baseline diagnosis was correct (which we call the “accurate diagnosis group”). However, in nine patients (40.91%), there was an erroneous baseline diagnosis (which we refer to as the “misdiagnosis group”). This initial mistake could be corrected during the evolutionary study.

We looked for baseline changes in the neuroimaging between the “misdiagnosis” and “accurate diagnosis” groups (Table 4) and found significant differences in the quantitative SPECT values between the two groups. In fact, we found significantly lower values in the “misdiagnosis group” with respect to the “accurate diagnosis group”. No significant differences were observed when comparing the means of the interstudy months of both groups (26.2 vs. 21.3, *p* = 0.5).

To quantify the effect in relation to time, we calculated the percentage of inter-study change, which was 31% at 12 months and 28–29% overall (Table 5). To determine whether, in cases of diagnostic error, there were greater or lesser changes in the images (i.e., a faster or slower progression of the MSA), the amount of net change between the two images (baseline versus evolutionary) was determined. This was performed globally and at 12 months for both groups (“misdiagnosis group” vs. “accurate diagnosis group”). In the “misdiagnosis group”, the progression of deterioration was less, and, therefore, less evident in their SPECT than that in the “accurate diagnosis group”. Moreover, a period of 12 months between the baseline and evolutionary study seemed to be very adequate for detecting possible changes.

We searched for the possible impact of asymmetry on the involvement of both striatal nuclei (as a reflection of asymmetry between cerebral hemispheres), with respect to the total change that occurred, the speed of the disease progression, and the possible emission of an erroneous diagnosis. We could not correlate the asymmetry in the involvement of both striatal nuclei with the total change experienced in the baseline (r = 0.39, *p* = 0.135) and final studies (r = 0.028, *p* = 0.927). Neither did they differ with respect to the speed of the disease progression for the baseline (r = 0.094, *p* = 0.728), final (r = 0.09, *p* = 0.758), or the difference between the two (r = 0.11, *p* = 0.696). Finally, we found no significant differences in issuing a misdiagnosis in terms of the mean scores for the baseline skewness (0.009 “accurate diagnosis group” vs. 0.001 “misdiagnosis group”, *p* > 0.05), final skewness (0.017 “accurate diagnosis group” vs. −0.008 “misdiagnosis group”, *p* > 0.05), and baseline–final difference (−0.013 “accurate diagnosis group” vs. 0.005 “misdiagnosis group”, *p* > 0.05).

## 4. Discussion

Our purpose was to describe and analyze the diagnostic effectiveness of performing serial brain SPECT studies of presynaptic dopamine transporters with [^123^I]Ioflupane for MSA.

Based on the results presented, we can affirm that performing repeated SPECT with [^123^I]Ioflupane was useful and effective in the diagnosis and follow-up of MSA. In our experience, instead of performing a single SPECT for the study of MSA, performing an evolving SPECT study improved both sensitivity (increasing from 50% to 75%) and PPV (increasing from 72.7% to 75%). Moreover, in those cases of initial (or baseline) MSA with an inconclusive SPECT study, 100% of them were diagnosed.

Based on the overall S/O index change, we calculated the progression of the MSA. Consequently, we were able to determine the amount of global striatal deterioration for the first time, which was 28–29% on average overall and 31% after one year of follow-up. These findings are consistent with the characteristic evolution of this disease, whose rapidly progressive course triggers severe patient deterioration and poor survival [1,2,4,19]. Knowing this amount of functional loss can help to understand the pathochrony of this disease, as already pointed out in some studies where SPECT was considered as a biomarker of progression [14,16]. 

In a previous cross-sectional study by our group, we observed that there was asymmetry in the initial involvement of both striatal nuclei, with the left one being greater [20]. However, in the present work, we were able to verify that, although both striatal nuclei significantly worsened over time, the speed of deterioration was similar in both nuclei. This was another first for the present study. However, the analysis of the degree of asymmetry (lateralization) in the striatal involvement did not seem to play an important role in MSA, since we could not establish an association of this asymmetry with the impact on the total changes undergone, either with the speed of the progression of the disease or the possible emission of an erroneous diagnosis. 

In our casuistry, the period in which the neurologists most frequently requested the evolutionary SPECT was 12 months. Based on our results, we believe it is appropriate to establish a cut-off of 12 months as the appropriate time to perform the evolutionary study for several reasons: (i) When analyzing the group of patients in which there was an initial misdiagnosis with respect to the group with a correct diagnosis, we found a greater diagnostic accuracy when using this cut-off. (ii) Given the percentage of functional loss mentioned above, this would allow us to distinguish it from other entities whose evolution is slower. (iii) When analyzing the changes in the SPECT, both qualitative and quantitative, there was a clear difference between the “misdiagnosis group” and the “accurate diagnosis group”, which would allow for establishing an early accurate diagnosis and, consequently, an early initiation of treatment.

**Figure 1:**
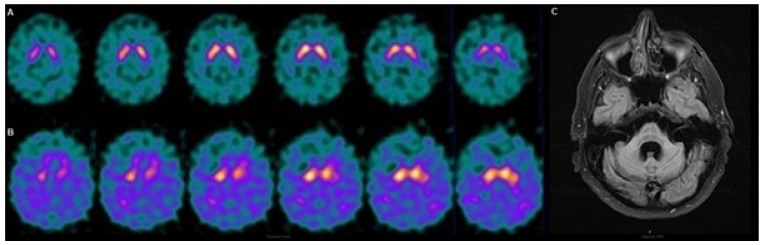
Follow-up of patient with MSA-C. A 62-year-old woman with MSA-C with dysautonomic, parkinsonian, and cerebellar symptoms, unresponsive to levodopa treatment. The evolutionary SPECT allowed the detection of bilateral striatal disease. (**A**) Anodyne basal SPECT. (**B**) Evolutionary SPECT at 12 months with noticeable bilateral striatal dysfunction. (**C**) MRI showed the characteristic “hot cross bun” sign in the cerebellum. The SPECT images are at 5 cm and the MRI image is at 10 cm.

This is especially important for distinguishing between the MSA-P and MSA-C subtypes. In a previous cross-sectional study by our group, qualitative SPECT assessments were found to be accurate in differentiating between the two subtypes. Given that, in this disease, diagnostic errors can occur in the first SPECT assessment (especially in the MSA-C subtype), a serial study would allow for the detection of false negatives or doubtful results and correctly establish the diagnosis [11,15,16,21]. As an example of this, we found in our casuistry a 62-year-old woman with dysautonomic, parkinsonian, and cerebellar symptoms, unresponsive to treatment with levodopa, and it finally turned out to be a case of MSA-C with bilateral striatal involvement (Figure 1). Moreover, since a higher rate of progression has been demonstrated in the P form with respect to the C form, an evolutionary study would make it possible to establish this differentiation with a greater accuracy and confidence [4,11,12,14,22,23].

It should be noted that most of our patients had MSA-C, so the percentage of annual deterioration obtained could be higher in cases of MSA-P. In this regard, we recommend that future studies should not only analyze MSA as a whole, but also differentiate separately between the two subtypes of the disease, especially taking into account that SPECT may have a clear difference in its diagnostic effectiveness between MSA-P and MSA-C, as our group observed in a previous cross-sectional study.

After performing the evolutionary SPECT, it was observed that the diagnosis based on the qualitative assessment of SPECT (performed by the nuclear medicine physician) remained without differences in both time periods, whereas the quantitative analysis did show significant differences, reflecting the progressive deterioration characteristic of MSA. On the other hand, the diagnostic suspicion initially established by the neurologist did not always coincide with the confirmatory diagnosis obtained with the evolutionary SPECT, so diagnostic reassignments had to be made. These reassignments were significantly related to the qualitative assessment of the SPECT, which provides greater value to the evolutionary or serial study. 

**Figure 2:**
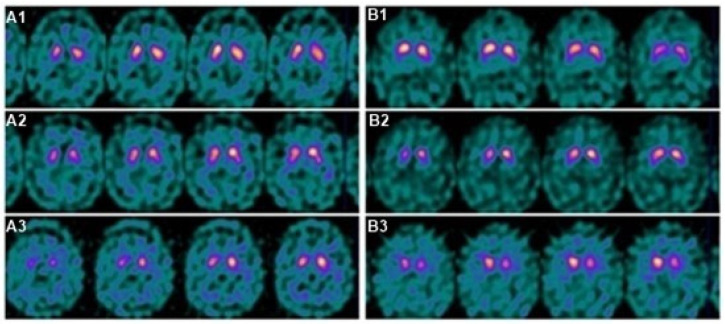
SPECT evolution of striatal involvement in two patients (**A**,**B**) with MSA. (**A**) Seventy-one-year-old male with MSA-P, studies performed at baseline (**A1**), 11 months (**A2**), and 3 years (**A3**). (**B**) Forty-eight-year-old male with MSA-C. SPECT at baseline (**B1**), 9 months (**B2**), and 2 years (**B3**). The SPECT images are at 5 cm.

When the changes between the baseline and evolutionary studies were studied, in the “misdiagnosis group”, the progression of the deterioration was slower, so the progression of the disease was more insidious. We believe that this slower progression was related to the fact that, in the baseline study, the changes in the neuroimaging were more subtle in the “misdiagnosis group” and, therefore, more difficult to detect (which led to a greater probability of making an erroneous diagnosis). Therefore, in the event of initial diagnostic doubt, the evolutionary study would allow us to confirm or rule out the existence of involvement (Figure 2). 

The weaknesses and strengths of this study can be presented in parallel, as both are closely related. One weakness could be that it was a single-center study (which diminishes its external validity). However, this gives it a strength, as all the neuroimaging tests were evaluated by the same physicians (two senior nuclear medicine specialists and one junior specialist, with the diagnosis being obtained by a group decision). If it had been carried out in several hospitals with different physicians, inter-observer bias would have to be considered. The sample size can be considered as a weakness, although we should not forget that MSA is a rare disease with a fatal prognosis. This makes patient recruitment very difficult and, above all, difficult to establish loyalty for a possible follow-up. For this reason, there are very few neuroimaging follow-up studies, with some being retrospective [15] or having a smaller sample size [13]. It could have been considered to recruit a cohort of healthy patients (as a control group) to study them comparatively with the MSA patients; however, no ethics committee could approve the administration of radionuclides in healthy patients. Undoubtedly, the greatest strength of this study is its longitudinal design, as this allowed for each subject to be his or her own comparison features. This lends a great robustness to the results, especially if we consider that the brain imaging studies were carried out under identical conditions, i.e., with the same SPECT device and the same physicians.

In our opinion, in order to obtain more information regarding the diagnostic effectiveness of [^123^I]Ioflupane SPECT for MSA, studies based on a larger number of patients and with longer follow-up times would be necessary. With this, it would even be possible to perform repetitive SPECT (at shorter intervals), in order to know with a greater accuracy which would be the most appropriate cut-off for indicating the performance of a new SPECT.

## 5. Conclusions

In conclusion, we can say: (i) the evolving SPECT study is useful in the diagnosis of MSA, with a higher diagnostic yield with respect to baseline SPECT; (ii) one third of global striatal function deteriorates at 12 months, so we believe it is more appropriate to perform the evolutionary study at that time point; and (iii) striatal deterioration evolves unfavorably globally, without a clear lateralization of involvement.

## Figures and Tables

**Table 1:** Demographic, clinical, and diagnostic characteristics.

N = 22 (100%)	
Subjects	▪ M 11 (50%) ▪ W 11 (50%)
Diagnosis	▪ MSA-probable 19 (86.36%) ▪ MSA-possible 3 (13.64%)
MSA type	▪ MSA-C 12 (54.55%) ▪ MSA-P 10 (45.46%)
Initial clinical suspicion	▪ MSA 18.2%, ▪ MSA-C 40.9% ▪ MSA-P 27.3%. ▪ Atypical parkinsonism 13.6%
Final clinical suspicion	▪ MSA-C 50% ▪ MSA-P 22.7% ▪ Atypical parkinsonism 18.2% ▪ Non-established 9.1%
Basal SPECT	▪ Pathological 50% ▪ Normal 31.8% ▪ Inconclusive 18.2%
Evolutionary SPECT	▪ Pathological 72.7% ▪ Normal 27.3% ▪ Inconclusive 0

N: Number of patients; M: Men; W: Women; MSA: Multiple System Atrophy; MSA-P: Multiple System Atrophy Parkinsonian Type; and MSA-C: Multiple System Atrophy Cerebellar Type.

**Table 2:** Quantitative analysis of both SPECT studies (basal and evolutionary).

S/O Indices	Basal SPECT	Evolutionary SPECT
Global S/O	$\bar{x}$ 1.43 (SD 0.25) [0.9–1.88]	$\bar{x}$ 1.29 (SD 0.2) [0.94–1.7]
Right S/O	$\bar{x}$ 1.437 (SD 0.26) [0.89–1.86]	$\bar{x}$ 1.29 (SD 0.21) [0.92–1.73]
Left S/O	$\bar{x}$ 1.43 (SD 0.25) [0.91–1.9]	$\bar{x}$ 1.28 (SD 0.2) [0.96–1.68]

S/O: Striatum/Occipital; $\bar{x}$: Average score; and SD: Standard deviation. The minimum and maximum scores are shown in square brackets.

**Table 3:** Diagnostic accuracy of basal and evolutionary SPECT.

	Basal SPECT	Evolutionary SPECT
S	50% (22.38–77.62)	75% (50.66–99.34)
E	50% (1.66–98.34)	33.33% (0–79.39)
PPV	72.73% (41.86–100)	75% (50.66–99.34)
PNV	27.27% (0–58.14)	33.33% (0–79.39)

S: Sensitivity; E: Specificity; PPV: Positive predictive value; and PNV: Negative predictive value. The 95% confidence interval is shown in parentheses.

**Table 4:** Changes between both quantitative SPECT assessments (basal–evolutionary) in relation to inter-study months for “misdiagnosis group” and “accurate diagnosis group”.

	Misdiagnosis Group	Accurate Diagnosis Group	*p* Value
Global S/O	0.155	0.505	*
Right S/O	0.131	0.511	**
Left S/O	0.150	0.508	*
Inter-study months	26.206	21.333	*p* > 0.05

S/O: Striatum/Occipital index; * *p* < 0.05; and ** *p* < 0.01.

**Table 5:** Amount of net deterioration according to SPECT: overall and 12-month assessment.

	Misdiagnosis Group N = 9		Accurate Diagnosis Group N = 13	
	Overall	12 Months	Overall	12 Months
Deterioration (%) Global S/O	21.5%	28.9%	34.1%	33.5%
Deterioration (%) Right S/O	22.7%	30.4%	33.7%	32.2%
Deterioration (%) Left S/O	20.3%	27.4%	34.5%	34.9%

N: Number of patients; and S/O: Striatum/Occipital index.

## Data Availability

Not applicable.
